# Indications, Measurements, and Complications of Ten Essential Neonatal Procedures

**DOI:** 10.1155/2023/3241607

**Published:** 2023-09-05

**Authors:** Zainab Bubakr Hamad Zubi, Ahmad Fadzil Bin Abdullah, Muhd Alwi Bin Muhd Helmi, Taufiq Hidayat Hasan, Noraida Ramli, Adam Al-Anas Bin Mat Ali, Mossad Abdelhak Shaban Mohamed

**Affiliations:** ^1^Department of Paediatrics, Sultan Ahmad Shah Medical Centre, International Islamic University Malaysia, 25200 Kuantan, Pahang, Malaysia; ^2^Department of Paediatrics, Kulliyyah of Medicine, International Islamic University Malaysia, 25200 Kuantan, Pahang, Malaysia; ^3^Department of Paediatrics, School of Medical Sciences, University Sains Malaysia, 16150 Kubang Kerian, Kelantan, Malaysia; ^4^Paediatric Clinic, Hospital Sultanah Bahiyah, 05460 Alor Setar, Kedah, Malaysia

## Abstract

About 10% of newborns require some degree of assistance to begin their breathing, and 1% necessitates extensive resuscitation. Sick neonates are exposed to a number of invasive life-saving procedures as part of their management, either for investigation or for treatment. In order to support the neonates with the maximum possible benefits and reduce iatrogenic morbidity, health-care providers performing these procedures must be familiar with their indications, measurements, and potential complications. Hence, the aim of this review is to summarise ten of the main neonatal intensive care procedures with highlighting of their indications, measurements, and complications. They include the umbilical venous and arterial catheterizations and the intraosseous line which represent the principal postnatal emergency vascular accesses; the peripherally inserted central catheter for long-term venous access; the endotracheal tube and laryngeal mask airway for airway control and ventilation; chest tube for drainage of air and fluid from the thorax; and the nasogastric/orogastric tube for enteral feeding. Furthermore, lumber puncture and heel stick were included in this review as very important and frequently performed diagnostic procedures in the neonatal intensive care unit.

## 1. Introduction

The vast majority of newborns adapt well to the transition from the intrauterine to the extrauterine life. Nevertheless, some need stabilization and sometimes even resuscitation [1]. About 10% of newborns require some degree of assistance to begin their breathing, and 1% necessitate extensive resuscitation [2]. Forty seven percent of death in children less than 5 years of age occur during the neonatal period with 75% of them occurs in the first week of life and approximately 30% in the first day. This considerably high rate of mortality can be reduced by providing skilled care for the neonates at birth and postnatally and providing immediate treatment for small and sick neonates [3]. Sick neonates are exposed to a number of invasive procedures as part of their management, either for investigation or for treatment. [4]. From the important factors that improved neonatal care are the miniaturization of blood samples needed for different blood investigations. The other factor is providing neonatal parenteral nutrition [5]. Different procedures are performed in the neonatal intensive care unit (NICU) with the placement of different lines, catheters, and tubes which are essential to provide nutrition, withdraw blood samples, monitor arterial and venous blood pressures, analyse blood gases, ventilate the lungs, and drain fluid and air from the thorax. Each procedure requires the prediction of specific measurements that are of paramount importance since incorrectly placed lines, catheters, and tubes can lead to severe adverse effects in already very fragile neonates. In order to support the neonate with the maximum possible benefits and reduce iatrogenic morbidity, health-care providers performing these procedures must be familiar with their indications, measurements, and potential complications. Hence, the aim of this review is to summarise ten of the main NICU procedures with highlighting of their indications, measurements, and complications. They include umbilical venous catheterization (UVC), umbilical artery catheterization (UAC), peripherally inserted central catheter (PICC), intraosseous line (IO), endotracheal tube (ETT), laryngeal mask airway (LMA), chest tube, nasogastric/orogastric tube (NGT/OGT), lumber puncture (LP), and heel stick.

A number of crucial general rules should be taken into account if any procedure is decided to be performed. These rules include considering alternative noninvasive procedure if applicable, obtaining family informed consent, a trained health care provider should perform the procedure in the presence of skilled operator for guidance and assistance, choosing the most appropriate devices, using aseptic techniques, wearing personal protective equipment as safety measures for the operator, pain evaluation and control via suitable recommended methods either pharmacological or nonpharmacological, monitoring of cardiorespiratory and thermoregulatory stability throughout the procedure and ensuring that the correct procedure for the correct patient on the correct side is being performed with documentation of all details of the procedure [6].

## 2. Umbilical Venous Catheterization (UVC)

### 2.1. Indications of Umbilical Venous Catheterization

The umbilical venous catheter is a reliable, easy, and life-saving commonly used procedure in the NICU [7]. It is used as an immediate postnatal emergency vascular access for fluid and treatment administration and for blood sampling. It provides a long-term central venous access in low-birthweight and critically ill infants for infusion of fluid, medication, parenteral nutrition, and hypertonic or hyperosmotic solutions. It is also indicated for exchange transfusion, blood and blood products transfusion, and monitoring of central venous pressure [8, 9]. Umbilical venous catheter is the primary postnatal emergency access that can be used till alternative peripheral or central venous access can be secured [6]. To minimize the overuse of UVC, Shahid et al. established guidelines standardizing its usage based on gestational age and severity of illness. According to their guidelines, UVC should be placed in all preterm infants ≤ 28 weeks. It should be avoided in infants ≥ 29 weeks unless the newborn is intubated and ventilated, FiO_2_ ≥ 40% on continuous positive airway pressure, unstable hemodynamically requiring inotropes or fluid bolus, or peripheral intravenous catheter (PIV) is difficult to establish [10]. It should be removed as soon as possible if not indicated but can be used up to 2 weeks if managed aseptically [11].

### 2.2. Size and Measurement of Umbilical Venous Catheterization

For infants weighing <3.5 kg, UVC size of 3.5 Fr is used, whereas 5 Fr catheters are used for infants weighing >3.5 kg [12]. Eight Fr catheter is recommended for exchange transfusion [9]. Double- and triple-lumen UVC are used when administration of incompatible solutions is required [6].

Several methods have been used for the estimation of the optimal measurements of UVC insertion length. The most widely used methods are Dunn's method, Shukla and Ferrara's method, Vali et al.'s formula, Verheij et al.'s formula, and Gupta et al.'s formula, as demonstrated in Table 1. The umbilical stump length should be added to the calculation. In Dunn's method, the shoulder-umbilical length is the distance between the top of the shoulder over the lateral end of the clavicle and a point vertically beneath it that is level with the centre of the umbilicus [13]. Shukla and Ferrara's method characterized by easy and rapid estimation of umbilical catheter length, particularly in the emergency situations [14]. As the Shukla formula is frequently associated with over-insertion of UVC, a revised formula has been proposed by Verheij et al. The revised formula is associated with less over-insertion of UVC without being complicated with a low-position UVC [15]. The Gupta et al. formula showed more accurate estimation of UVC length than the Shukla formula in neonates with different body weights [16].

### 2.3. Position of Umbilical Venous Catheter

The optimal position of the UVC tip is at the junction between the inferior vena cava and right atrium, or at least well into the ductus venosus [19]. The ideal UVC tip position is at the level of T8-T9, but it could be anywhere between T7 and T10, high if above the body of T8 and low if below the body of T9 [20, 21]. Umbilical catheter tip position should be confirmed radiologically after insertion, regardless of the method used to estimate placement length. Anteroposterior chest-abdominal radiograph and lateral X-rays are commonly used for confirmation of catheter tip position. However, it has been suggested to use ultrasound, once available, as the modality of choice for the determination of catheter tip position [20, 22, 23]. The ultrasound technique has been described as more accurate, less harmful, and provides more information than the X-ray image, which carries the risk of exposure to radiation and influenced by a child's movement, an overlying temperature probe, and the presence of congenital defects [23]. Echocardiography/aortography has also been proposed as a superior modality to radiography in the determination of umbilical catheter tip position [24].

### 2.4. Complications of Umbilical Venous Catheterization

Although the UVC is an easy, frequently used, and life-saving procedure, it may induce serious complications with a significant morbidity and mortality [25]. Complications of the UVC include infection, sepsis, thrombosis [26, 27], necrotizing enterocolitis [28], extravasation of parenteral nutrition, and drugs to the liver parenchyma [29], hepatic collections with liver capsule rupture and ascites [30], portal hypertension and hepatic atrophy secondary to portal vein thrombosis [31] and severe hepatic injury secondary to malposition of UVC into the portal circulation [32].

Although rare, some severe complications of UVC have been reported in the literature. They include UVC rupture [7], perforation of the umbilical vein with haemorrhage into the peritoneal cavity [33], pulmonary oedema, pulmonary haemorrhagic infarction, and hydrothorax which resulted from inappropriate positioning of the tip of the UVC into the pulmonary vein [34]. Inappropriately placed UVC within the heart induces cardiac arrythmia [35], thrombotic endocarditis [36], pericardial effusion, and cardiac tamponade [37].

## 3. Umbilical Arterial Catheterization (UAC)

Umbilical artery catheterization has become a commonly performed procedure in the NICU and in the care of extremely preterm neonates [38, 39]. It should be inserted only in critically ill infants [6]. It should be removed as soon as possible when no longer needed and should not be kept inserted for more than 5 days [11].

### 3.1. Indications of Umbilical Arterial Catheterization

Umbilical arterial catheterization is indicated for frequent or continuous measurement of arterial blood gases, continuous monitoring of arterial blood pressure, angiography, and resuscitation (umbilical venous line is the first choice). In emergency situations, UAC can be used for administration of medications, fluids, and blood products, provided that no alternative intravascular line is available. It is also indicated as a temporary route for infusions, parenteral nutrition, and medications, particularly in extremely low-birthweight neonates. An umbilical artery catheter should not be used for the administration of vasopressors, indomethacin, boluses of calcium, and anticonvulsant [9]. According to Shahid et al.'s standardizing guidelines, UAC is recommended to be placed in all preterm infants ≤ 26 weeks and in infants > 26 weeks who are intubated and ventilated, or FiO_2_ > 40% on continuous positive airway pressure, or the infant is hemodynamically unstable, needing fluid bolus or inotropes [10].

### 3.2. Size and Measurement of Umbilical Arterial Catheter

For infants <1.5 kg, UAC size 3.5 Fr is used, whereas a 5 Fr catheter is used for infant >1.5 kg [9]. Multiple methods and formulas have been proposed for measurement of UAC insertion length. The main methods that are used are Dunn's formula, Shukla and Ferrara's method, Wright's formula, Gupta et al.'s formula, and Vali et al.'s formula as shown in Table 1. It is important to add the length of the umbilical stump to the calculation. There is no consensus regarding the best method for accurate catheter positioning [6]. Wright's formula is associated with more accurate placement and less overinsertion of UAC than Dunn's formula, particularly in infants weighing less than 1000 g [17]. Kumar et al. demonstrated that there is no universal formula for accurate measurement for the placement of UAC; however, Wright's formula is the closest in neonates with different birth weight [40]. Gupta et al.'s formula provided a better estimation of UAC length in infants with different birthweights in comparison to Shukla and Wright's formula [16].

### 3.3. Position of Umbilical Arterial Catheter

Based on the position of its tip in the aorta, umbilical artery catheter is divided into high-position and low-position UAC. In high-position UAC, the tip is located in the descending thoracic aorta above the diaphragm at the level of the seventh to ninth thoracic vertebrae (T7 to T9), avoiding the origins of the mesenteric, celiac, and renal arteries. The tip of low-position UAC is located in the abdominal aorta at the level of the third to fifth lumber vertebrae (L3 to L5), at or below the aortic bifurcation, and below the origin of the abdomen's major blood supply. It has been recommended to place the tip of the UAC in a high position [41]. After UAC insertion, it is essential to confirm UAC tip position radiologically through performing anteroposterior chest-abdominal radiograph and lateral X-ray. Ultrasound and echocardiography are proposed as safe and reliable methods for confirmation of umbilical catheter tip position [20, 22, 23].

### 3.4. Complications of Umbilical Arterial Catheterization

Umbilical artery catheterization is associated with significant complications such as thrombosis and thromboembolism, which occur either from a blood clot or air in the infusion system [8]. Umbilical arterial catheterization-induced abdominal aortic thrombosis can cause necrotizing enterocolitis [42], congestive heart failure [43], mycotic aortic aneurysm [44], multiple aortic aneurysms, and aortic pseudoaneurysms [45, 46]. As aortic thrombosis is a common complication of UAC insertion, it has been recommended that any infant who experiences UAC insertion should be screened for abdominal aortic thrombosis upon UAC removal [47]. Long-term follow-up for hypertension, renal abnormalities, and leg-growth disturbances should be considered in any infant with proved aortic thrombosis post UAC removal, as these sequelae have been reported on long-term follow-up [48]. Umbilical arterial catheterization-related thromboembolism may affect vessels other than the aorta such as the mesenteric artery which causes necrotizing enterocolitis [49], and renal artery which is complicated by neonatal hypertension [50, 51]. Other reported complications of the UAC placement include bleeding following accidental disconnection or overheparinization [8], vasospasm or blanching of the lower limb [52], neonatal infection and sepsis [53], intestinal perforations [54], malposition of the UAC into the subclavian, gluteal, and renal arteries, and the coeliac axis [55], and factitious hypernatremia and hyperkalaemia [56].

Umbilical arterial catheter placement is associated with rare complications such as twisting of the UAC in the aorta, which increases the risk of thrombus formation and perforation [57], Wharton jelly embolism [58], herniation of the appendix through the umbilical ring [59], neonatal bladder injury, rupture, and ascites [60], flaccid paraplegia resulted from spinal cord ischemia as a result of vasospasm or embolism of the Adamkiewicz artery [61], spasm of the inferior gluteal artery with a subsequent gluteal gangrene and peroneal nerve palsy [62], malposition into the femoral artery with a subsequent lower limb ischemia [63], lower limb ischemia and amputation [64, 65], umbilical artery perforation complicated with severe haemorrhagic shock, renal failure, and severe periventricular leukomalacia, and neonatal death [66], and refractory hypoglycaemia, which has been attributed to direct stimulation of the pancreas via streaming of glucose into the pancreas with a net effect of hyperinsulinemia and hypoglycaemia [67].

## 4. Percutaneous Central Venous Catheterization (Peripherally Inserted Central Catheter (PICC))

Percutaneous central venous catheterization is indicated when long-term venous access is anticipated. Its placement is recommended when the duration of intravenous therapy is expected to exceed six days [11]. It is used for administration of fluids, hypertonic solutions, medications including vasopressors and parenteral nutrition, and for monitoring of central venous pressure [68, 69]. Frequent blood sampling is not a primary indication for PICC insertion where larger-lumen catheters are used for blood draws without risk of clotting [8]. The catheter can be introduced through either the upper or lower limb. The more appropriate veins are the basilic vein in the upper limb and the greater saphenous vein in the lower limb. Axillary or femoral veins are infrequently used, and the cephalic vein should be avoided owing to the difficulty of central placement through it [52].

### 4.1. Size and Measurement of Peripherally Inserted Central Catheter

A peripherally inserted central catheter of size 1 F is used for neonates weighing less than 1 kg, and a 2 F catheter is used for neonates with a body weight more than 1 kg [6]. The length of PICC to be placed through the upper body insertion is determined by measuring the distance from the insertion site through the course of the used vein to the head of the clavicle on the right side and down to the right third intercostal space just to the right of the sternum. For PICC placed through lower limb insertion, the required insertion length is equal to the distance measured from the insertion site through the course of the used vein till the level of the xiphoid process [52].

### 4.2. Position of Peripherally Inserted Central Catheter

The optimal tip position of a PICC inserted through the upper limb veins is in the superior vena cava (SVC) at T3-T5 level. For lower limb-inserted PICC, the ideal tip position is in the inferior vena cava (IVC) between the diaphragm and the right atrium at T8-T10 level [70]. The general consensus is that the tip of the PICC should not be placed within the right atrium or allowed to migrate to the heart [8, 68]. Chest radiography including a portion of the upper arm and neck is used for verification of PICC tip position if the insertion site is in the arm, and includes the abdomen if the PICC is inserted through a lower extremity [70]. The anteroposterior view is the most commonly used view for confirmation of PICC tip position; however, adding on lateral view is more reliable in the detection of catheter tip malposition, particularly in the lower extremity-inserted PICC [68]. As patient position and movement significantly affect PICC tip position, it has been recommended that radiography should be taken with an extremity in a position that produce catheter tip in a close proximity to the right atrium. It has also been suggested to take the radiography while the infant be positioned as he or she would comfortably spend most of the day as that will show the PICC tip position in the majority of the time [68]. Although Point-of-Care Ultrasound (POCUS) has been suggested as a low-risk modality for verification of PICC tip position, it has been recommended that its use should be a complement but not a replacement to conventional radiography [71].

### 4.3. Complications of Peripherally Inserted Central Catheter

Although the PICC provides the mainstay of long-term, stable venous access, its complications remain a concern. Peripherally inserted central catheter is associated with increased risks of catheter-related bloodstream infection [72], thrombosis [73, 74], extravasation and intrahepatic collections of hyperalimentation [75], massive consolidation of the upper lobes of both lungs [76], and plural effusion [77]. From the unusual complications of PICC, an embolism of fragment of the catheter tip into the peripheral branch of the left pulmonary artery, perforation of the pulmonary artery peripheral branch [78], focal neurological manifestations in a form of tonic-clonic movement of the lower limb induced by inadvertent placement of PICC in the ascending lumber vein [79], oliguria due to malposition of the catheter tip into the renal vein with a subsequent vein occlusion or renal damage by solutions administered through the renal vein [80], cardiac arrythmia [81], cardiac tamponade, and death [82, 83].

## 5. Intraosseous (IO) Infusion

It is an emergency vascular access for the infusion of fluids and medications if other venous accesses cannot be established [84]. It is considered to be a reasonable alternative to the umbilical venous catheter, the primary emergency vascular access in neonates, if UVC placement failed or cannot be established [1, 85]. It is recommended to be placed if venous access cannot be secured within three attempts and within 90 seconds [9]. Intraosseous catheter can be used both in preterm and full-term neonates [86]. All intravenous medications can be administered through the intraosseous infusion [87]. The cortex overlying the metaphysis of the long bone is thin and easy to be penetrated to reach the medullary cavity which is connected to the systemic circulation. Due to the noncollapsible nature of medullary cavity veins during hypovolemia and shock, they serve as an entry channel of administered fluids and medication, via IO, to the central circulation [88]. Intraosseous line should be removed once an alternative venous access can be secured [89].

### 5.1. Site of Insertion of Intraosseous Catheter

The preferred insertion site for neonate is the proximal tibia [85]. The targeted penetration point of the proximal tibia is at its flat anteromedial surface, 1 to 2 cm below and 1 cm medial to the tibial tuberosity. If there is difficulty in palpation of tibial tuberosity, the flat medial surface of the tibia 1.5 to 2 cm distal to the patella serves as the estimated penetration site [8]. The preferred second site in the infant is the distal femur [9]. The distal femur penetration site is 1 to 3 cm above the external condyles in the anterior midline [8]. The humerus has been suggested as a safe alternative site to the tibia for intraosseous needle placement; however, the conducted study was limited to a sample of neonatal cadavers. The suggested penetration site is at the greater tubercle 9.5 mm – 11.1 mm from the acromion [90]. Proximal humeral head and distal femoral end were suggested, in another study undertaken on 20 stillborns, as possible alternatives to the proximal tibia [91]

### 5.2. Complications of Intraosseous Catheter

In a recently published prospective study conducted on 145 full-term and 16 preterm infants, intraosseous line was described as a fast, feasible, and safe emergency vascular access with a very low rate of complications. The intraosseous catheter-associated complications have been divided into severe and minor complications. The severe complications occur in 6% of the patients and include necrosis, fracture, broken IO needle, osteomyelitis, soft tissue infection, and perfusion problems, whereas minor complications occurred in approximately 30% including misplacement in soft tissue, mild paravasation, healing deficiency, and local swelling [84]. Similar results have been reported by Ellemunter et al. who reported that the rate of complications was low and that the main complications observed were needle dislocation and malfunctioning, extravasation, subcutaneous necrosis, and hematoma [86]. Intraosseous needle insertion-related rare complications include lower limb amputation secondary to either compartment syndrome or tissue necrosis caused by calcium infusion [92, 93].

## 6. Endotracheal Tube (ETT)

Endotracheal intubation is a common procedure in the NICU and delivery room. Neonatal endotracheal intubation is indicated in resuscitation if the positive pressure ventilation (PPV) is ineffective with heart rate remains less than 100 beat/minute or prolonged PPV or chest compressions are required. It is also indicated for direct suction of thick secretions or meconium obstructing the trachea, for administration of surfactant or other medications, and for stabilization of neonates with congenital diaphragmatic hernia or extremely low birth weight [85, 94].

### 6.1. Size of Endotracheal Tube

Appropriate ETT size minimizes trauma, airway resistance, and excessive leakage around the tube [95]. Approximate endotracheal tube size is determined based on the birth weight and gestational age as illustrated in Table 2 [85].

### 6.2. Insertion Depth of Endotracheal Tube

Different methods have been used to estimate the proper insertion depth of ETT as given in Table 3. In the rule of 7-8-9, beyond 7 cm, for each additional one kilogram of body weight, one centimetre is added to determine the tip-to-lip distance [96]. The 7-8-9 rule has been found to be associated with an overestimation of tubal length if the infant weight is less than 1500 grams or more than 2500 grams [97, 98]. The American Academy of Paediatrics/American Heart Association's formula is a simplified form of the 7-8-9 rule [97].

A gestation-based guideline table for ETT length in neonates has been provided by Kempley et al. as demonstrated in Table 4. The table included the relationship between gestational age, weight, and endotracheal tube length, which is the initial length that should be confirmed radiologically [98].

The optimal placement of ETT is essential to deliver adequate ventilatory support and reduce the risk of complications. The ideal endotracheal tube tip position in neonate has been shown radiologically to be at the body of the first thoracic vertebra (T1), which was recommended as the more precise standard reference point in preference to either the medial clavicular end or the supracarinal distance, or the combination of the two. The position of the carina varies between T3 and T5 [101]. However, the acceptable ETT tip position is range from the upper border of the first thoracic vertebra and the lower border of the second thoracic vertebra [102]. Chest X-ray is the gold standard method for verification of ETT position. Ultrasound was suggested as a fast and effective tool for determination of the appropriateness of ETT position in neonates; however, it needs more studies to be established [103].

### 6.3. Complications of Endotracheal Intubation

Endotracheal intubation is a painful and distressing procedure that is not free from adverse effects [104]. A number of complications related to endotracheal intubation are described in the literature. These complications resulted from malpositioning or displacement of the tube, tubal obstruction by thickened secretions, accidental tube extubation, and prolonged intubation. ETT-related complications can be either localized to the respiratory system or systemic complications [105]. They were classified into severe and nonsevere complications. Severe complications include cardiac arrest, severe desaturation (≥20% decrease in SpO2), direct airway injury, oesophageal intubation with delayed recognition, vomiting associated with aspiration, hypotension necessitating treatment, laryngospasm, malignant hyperthermia, pneumothorax or pneumomediastinum, and death. Nonsevere complications include mainstem bronchial intubation, oesophageal intubation with immediate recognition, vomiting without aspiration, hypertension requiring medication, oral or airway bleeding, lip trauma, gum or oral trauma, medication error, arrhythmia, and pain and/or agitation requiring additional medication and causing delay in intubation [106, 107]. Endotracheal intubation is also associated with respiratory and systemic infections [108]. Pulmonary atelectasis is a reported serious complication of endotracheal intubation [109]. Subglottic stenosis is a late sequelae for endotracheal intubation [110, 111]. Numerous complications in the oral cavity are caused by tracheal intubation during oral development such as alveolar or palatal grooving, palatal deformation, defective development of enamel, tooth malformation, displacement of tooth germ, eruption sequence, crossbite, oral commissure defect, temporomandibular joint injury, tongue injury, and incorrect pronunciation [112]. The possibilities of complications increase with an increasing number of intubations attempts and are reduced with use of paralytic medications and intubator training [106, 107].

## 7. Laryngeal Mask Airway (LMA)

Laryngeal mask airway has been recommended as an alternative route to provide airway control and ventilation if bag-mask ventilation (BMV) is ineffective and tracheal intubation is failed or not feasible in newborns delivered at ≥34 weeks gestational age or their weights are more than 2 kg [113, 114]. It is indicated in babies with difficult intubation including isolated upper airway or craniofacial malformations, in syndromes such as Pierre-Robin or Cornelia de Lange syndromes, or with airway malformations like laryngeal clefts [115]. Laryngeal mask airway can be used as an effective airway interface for resuscitation in the delivery room and neonatal intensive care unit, and during transport, provided that adequately trained providers are available [116]. There is a lack of evidence to support the use of LMA in newborns less than 34 weeks gestations or less than 2 kg in body weight [113]. Insufficient evidence is available with regards to the routine use of LMA for surfactant and epinephrine administration [115]. Laryngeal mask airway was superior to BMV in terms of shorter resuscitation and ventilation times and was associated with more chance to avoid intubation with its use. Its effectiveness was comparable to endotracheal intubation [117]. Its use is also associated with less admission to the neonatal intensive care unit and reduce the length of hospitalization [118]. Prolonged ventilatory support using LMA for 3 days has been reported in a preterm neonate after many unsuccessful attempts of intubation [119]. Four days of laryngeal mask airway-mediated ventilatory support were reported in a neonate with Pierre-Robin syndrome [120] and in another with airway obstruction and Treacher Collins syndrome [121].

All neonatal LMAs are size 1 and are used for infants up to 5 kg, except Air-Q disposable LMA which is available at size 0.5 and is used for infants less than 4 kg [9].

### 7.1. Complications of Laryngeal Mask Airway

Few adverse effects are associated with LMA placement with an overall adverse effect rate of 11.5%, and no major morbidity was reported with its use [122]. The reported adverse outcomes include vomiting, regurgitation, gastric aspiration, gastric distention, laryngospasm, and bronchospasm [123–126]. It may induce soft tissue trauma to the epiglottis, uvula, and tongue [117]. An upper oesophageal lesion was induced by LMA resuscitation in an extremely low-birth-weight infant [127]. Laryngeal mask airway obstruction by supralaryngeal mucus plug was reported in a preterm infant with tracheoesophageal fistula [128].

## 8. Intercostal Catheter/Drain (Chest Tube, Thoracostomy)

The intercostal catheter is used for drainage of intrathoracic collections which could be air (pneumothorax) or fluid (plural effusion) including lymph (chylothorax), blood (haemothorax), pus (empyema), and oozing from the surgical site [21, 129–131].

### 8.1. Insertion Site and Length of Chest Tube

In both preterm and full-term neonates and at both thoracic sides, the Buelau position is suitable and safe for chest tube insertion as no other organs or structures apart from lung parenchyma are found. Buelau position takes place in the anterior to midaxillary line between the 4th or 5th intercostal space above the margin of the ribs [132]. It is advisable to insert the chest tube tip posterior to the lung for fluid removal from the plural space, whereas pneumothorax will be effectively drained if the tip of the chest tube is placed anterior to the lung, which can be achieved by inserting the tube close to the anterior axillary line with the direction of the tip anteriorly and towards the xiphisternum but away from the breast tissue [21]. The insertion length for the chest tube is 2 to 3 cm in small preterm infants and 3 to 4 cm in full-term infants [8]. The drainage procedure can be performed using either the traditional chest tube or the pigtail catheter, as they have comparable safety and effectiveness [133]. Pigtail catheter is considered to be a fast, easy, safe and effective alternative to traditional chest tube in premature infants [134]. After chest tube insertion, immediate anteroposterior and lateral chest radiography should be obtained to ascertain placement and check for residual fluid or pneumothorax [9]. The tip of the tube and all side holes should be located within the pleural space [135]. Point-of-Care Ultrasound-guided chest tube insertion is associated with a high success rate and a low risk of complications [71]

### 8.2. Complications of Chest Tube

The placement of a chest tube is an invasive, life-saving procedure, but it is associated with a risk of significant morbidity. Dislodgement and dysfunction are minor complications of chest tube insertion, whereas its major complications are infection and thoracic organ and structure injury including lung laceration and perforation, pleural effusion, pericardial perforation, phrenic nerve injury, and diaphragmatic paralysis [134, 136–138].

## 9. Nasogastric/Orogastric Tube

Neonatal enteral feeding can be achieved through a feeding tube that passes either through the nose (nasogastric tube) or through the mouth (orogastric tube) to the stomach or upper small intestine [139]. A nasogastric/orogastric tube is indicated in preterm infants less than 34 weeks gestation due to inadequate coordination of sucking and swallowing, neurological immaturity, and respiratory compromise, and in sick neonates able to tolerate enteral feeds but are unable to feed for themselves [140, 141]. Nasogastric/orogastric tube used to provide complementary feeding for preterm infants unable to take full feeding. It is used for gastric decompression, administration of medications, and gradual weaning from parenteral nutrition [142, 143]. It is also indicated in infants with impairment of suck/swallow coordination due to encephalopathy, hypotonia, and maxillofacial abnormalities [6]. It is placed in cases experiencing severe respiratory distress and used to prevent pulmonary aspiration of gastric contents and measurement of gastric residuals. It is inserted and removed immediately in full-term infants to rule out obstruction of the posterior naris or oesophageal atresia [142]. There is no conclusive recommendation regarding the superiority of either the nasogastric or orogastric tube over each other [144]. No difference was found between the nasogastric tube and the orogastric tube in terms of time to regain body weight, occurrence of adverse effects, and time to full feed. Nevertheless, practising of nasogastric tube was superior to orogastric tube with less chance of displacement. [140]. The nasogastric tube is easier to secure to the face than the orogastric tube [139]. The orogastric tube can be easily displaced as it can loop inside the mouth [139]. An orogastric tube has been suggested to be placed in neonates less than 2 kg in preference to NGT due to associated pulmonary compromise [145]. The orogastric tube is selectively used in cases of nasal blockage by nasal atresia and nasal cannulas or continuous positive airway pressure tubes; respiratory intolerance or breathing problems with the nasogastric tube, which manifested by apnoea and bradycardia [143].

### 9.1. Size and Measurements of Nasogastric/Orogastric Tube

Infant feeding single-lumen tube size 3.5 F or 5 F is used for infants <1000 g and size 5–8 F is used for infants ≥ 1000 g. For decompression, a dual-lumen vented Replogle tube with size 6, 8, or 10 F is used. Stylet is not recommended to be used in neonates [9].

Several measurement techniques are used to anticipate the insertion length of the NGT/OGT. The conventional measurement technique is the distance from the tip of the nose to the earlobe and then to the xyphoid process (NEX). Although this is the most commonly used technique, no research basis has been found for its use [146]. With using of NEX technique, the tube was found by Ziemer and Caroll and Tedeschi et al. to be either just passing the cardiac sphincter or located at the lower oesophagus and associated with increasing apnoea, desaturation, and bradycardia during feeding. Based on their observations, they developed another more accurate measurement from the tip of the nose to the earlobe to a point midway between the xiphoid and the umbilicus (NEMU) [147, 148].

In very low-birth-weight infants, the minimum insertion length for the orogastric tube was estimated based on the body weight as illustrated in Table 5. This estimated insertion length ensures the adequate positioning of the tube and reduces the number of tubes that are inappropriately placed in the oesophagus [149]

In 2011, an age-related, height-based (ARHB) equation specific to neonatal NGT length measurement has been developed by Ellett et al. who strictly recommended avoiding the use of NEX but suggested either ARHB for NGT, or NEMU for NGT/OGT as they are more accurate [150, 151]. NGT insertion length (cm) = 1.950 cm + 0.372 × length (cm), as demonstrated in Table 6 [150].

In 2012, a weight-based formula has been proposed by Freeman et al. for the estimation of gastric tube insertion length. Orogastric tube insertion length (cm) = [3 × weight (kg) + 12], whereas nasogastric tube insertion length (cm) = [3 × weight (kg) + 13] [152]. Application of a weight-based formula showed 84% correct tube placement, which was clinically and statistically significant and described as an improvement in the correct placement of the gastric tube [153].

Among the different measurement techniques, NEMU has been shown to be the most accurate [154]. The abdominal radiograph is the only certain way to determine the accurate placement of the nasogastric tube after insertion using the previous measurement techniques [155].

### 9.2. Complications of Nasogastric/Orogastric Tube

Nasogastric tubes may contaminate infant feeds even within the first 24 hours of use [156]. It is associated with partial nasal obstruction and increased nasal and total airway resistance [157]. Newborns less than 2 kg experienced significant pulmonary compromise with nasogastric tube placement. The compromise was in a form of diminished minute ventilation and respiratory rate and increased pulmonary resistance, resistive work of breathing, and peak transpulmonary pressure change [145]. The orogastric tube is associated with a transient increase in total and reduced haemoglobin and cerebral blood volume. [158]. Quick insertion of the feeding tube is associated with bradycardia and desaturation [159]. It has been estimated that 59% of the feeding tubes are placed incorrectly [160]. Mispositioned NG/OG tubes can cause numerous complications such as gastroesophageal reflux, aspiration, failure to gain weight, and diarrhoea [150, 161]. It can also cause severe complications such as oesophageal and stomach perforation [162, 163], perforation of the posterior pharynx [164], and chylopneumothorax secondary to oesophageal perforation with subsequent misplacement of the tube tip into the right pleural space [165]. Tearing of the nasogastric tube with a persistent of the missing part in the gastric cavity with its end near the gastric outlet has also been reported as a complication of NGT placement [166].

## 10. Lumber Puncture (LP)

Lumber puncture is one of the well-known procedures in the clinical paediatric. Its main indication in the neonatal period is suspected central nervous system infection, whether it is meningitis, encephalitis, or congenital infection. Its other indications include the evaluation of effectiveness of antimicrobial treatment, diagnosis of metabolic diseases, diagnosis of intracranial haemorrhage, diagnosis of leukaemia infiltrating the central nervous system, administration of intrathecal therapy, drainage of cerebrospinal fluid in nonobstructive hydrocephalus, and installation of contrast media for spinal cord imaging [8].

### 10.1. Needle Size for Lumbar Puncture

The standard gauge needle for LP in neonates is 22 G and 3.5 cm long needle [167]. However, a recent observational study showed that the use of a 25-gauge needle associated with a comparable success rate and less traumatic LP as compared to a 22-gauge needle [168].

### 10.2. Site and Depth of Lumber Puncture

Since the lower end of the spinal cord in neonates is at the level of the body of the third lumber vertebra (L3), the LP must be performed below the L2-L3 interspace. Optimal positioning of the neonate in either the sitting position or the lateral recumbent position is of paramount importance for the success of the procedure. An imaginary line joining the two uppermost points of bilateral posterior superior iliac crests will intersect in the midline just superior to L4. The interspaces between the third and fourth lumber vertebra (L3-L4) or between the fourth and fifth lumber vertebra (L4-L5) are the suitable places for LP in neonates [169]. The direction of the needle should be cephalad toward the umbilicus in the lateral recumbent position and slightly caudal in the sitting position [169]. To avoid deep insertion of the needle which causes traumatic LP, the insertion length has been determined in different ways as demonstrated in Table 7. A recently published work has advised the use of the prone position in preterm and low-birth weight infants as it is associated with higher overall success rate and higher success rate from the first attempt, meanwhile it is more comfortable with low incidence of adverse effects [170]. NeoCLEAR open-label, 2 × 2 factorial, randomised controlled trial, the largest trial investigating paediatric lumbar puncture, has been conducted in 21 UK neonatal and maternity units on infants with ages ranging from 27 to 44 weeks corrected gestational age and weighing 1 kg or more. This large multicentre trial has recently published that the sitting position is superior to the lying position in view of higher success rate, more comfortable, and better tolerated in terms of oxygen saturation and heart rate [171].

### 10.3. Complications of Lumber Puncture

Despite potential adverse effects, LP is not associated with increased mortality [177]. Traumatic or failed LP accounts for 30-50% of cases [178]. The lumber puncture success rate is approximately 60%, and prematurity is not associated with a higher risk of LP failure. The risk of oxygen desaturation with the procedure increases in low gestational age and mechanically ventilated neonates, whereas the risk of intraventricular haemorrhage increases in neonates with risk factors of bleeding. Lumber puncture-related bradycardia is self-resolving [179]. The association between lumber puncture and iatrogenic meningitis is controversial between the different studies [180–182]. Repeated lumber puncture in premature infants carries the risk of lumbar epidural abscess and vertebral osteomyelitis [183]. Cerebellar herniation is a rare complication of lumber puncture in neonates [184]. Spinal epidural hematoma complicated with paraplegia in previously undiagnosed haemophilia infant is from the unusual complications of lumber puncture [185]. Paraplegia was also reported in premature infant due to a conus medullaris lesion complicating lumber puncture [186]. Epidermoid spinal cord tumour is a late complication of LP [187].

## 11. Heel Stick/Heel Lance/Heel Prick

A heel stick is a pinprick puncture in one heel of a neonate in order to get a blood sample. It is a minimally invasive procedure performed repeatedly in the neonatal intensive care unit as a part of routine care of sick neonates. Capillary heel sampling is suitable for routine laboratory tests, frequent bedside monitoring of blood glucose, bilirubin testing, and blood gas analysis provided that an arterial line is not needed. It is the conventional blood collection technique for neonatal screening tests for phenylketonuria, hypothyroidism, cystic fibrosis, Duchenne muscular dystrophy and haemoglobinopathies. However, capillary heel sampling is not recommended for analysis of blood culture, coagulation profile and for tests requiring samples of blood more than 1 ml [188–191]. The heel stick is described as one of the painful procedures to the newborns [192]. Multiple interventions have been described in the literature to reduce heel stick-associated pain such as breast feeding during the procedure [193], skin-to-skin positioning before and during a heel stick [194], swaddling and heel warming prior to the puncture [192, 195], gentle massage of the leg before the heel stick [196], mechanical vibration [197], use of sucrose [198], and facilitated tucking and oral dextrose [199].

### 11.1. Site and Depth of Heel Stick

The recommended site for heel puncture is the most medial and lateral parts of the plantar surface of the heel with a maximum depth of 2.4 mm, not on the posterior curvature of the heel or through previous puncture sites, to avoid puncture of the calcaneus and the development of osteochondritis. The most lateral part is determined by a line that starts from the midway between the 4th and 5th toes and extends parallel to the lateral aspect of the heel, whereas the most medial part is medial to a line that runs from the middle of the great toe parallel to the medial surface of the heel [200]. Heel stick can be performed either through conventional manual lancets or using automated heel-lancing incision devices. The automated lancets are available in different sizes for neonate >1.5 kg (incision depth of 1 mm and length of 2.5 mm), for neonate <1.5 kg (incision depth of 0.85 mm and length of 1.75 mm), and for neonates < 1 kg (incision depth of 0.65 mm and length of 1.45 mm) [191]. Excessive squeezing should be avoided to limit infant pain and prevent sample compromise [191].

### 11.2. Complications of Heel Stick

Heel stick is a well-established, simply performed procedure in the neonatal care; however, it is not risk-free. The adverse effects that have been reported with heel stick are bruising, which is caused by excessive or prolonged squeezing [201], local infection at the puncture site complicated with suppurative inguinal lymphadenitis [202], ischemic necrosis of the foot skin [203], calcaneal osteomyelitis [204, 205], staphylococcal scalded skin syndrome [206], and calcified nodules on the heel induced by repeated heel sticks [207].

## 12. Conclusion

The physiologic and anatomic uniqueness of a newborn baby, particularly preterm neonates, requires a special approach to allow a smooth transition from the intrauterine to newborn life. Although this transition is uncomplicated in most of neonates, yet there are some of them require support in varying degrees at birth. This support may necessitate performing very critical and life-saving procedures. Ensuring adequate awareness about the neonatal procedure is essential to deliver the optimal care of sick neonates and reduce the possibility of occurrence of undesired adverse effects.

## Figures and Tables

**Table 1:** Methods of estimation of optimal measurements of umbilical venous and arterial catheters' insertion length.

Method	UVC	UAC	Reference
Dunn's method (1966)	Uses shoulder to umbilicus length and a nomogram to determine the insertion length	Uses shoulder to umbilicus length and a nomogram to determine the insertion length	[13]
Shukla and Ferrara method (1986)	UVC length (cm) = [3 × BW (kg) + 9]/2 + 1	UAC insertion length (cm) = 3 × BW (kg) + 9	[14]
Wright's formula (2008)	No proposed formula for UVC	UAC insertion length (cm) = (4 × BW [kg]) + 7	[17]
Vali et al.'s formula (2010)	UVC insertion length equal to the measurement from the umbilicus to the midxiphoid-to-bed distance, measured lateral to the baby	UAC length (cm) = 1.1 × (xiphoid–ASIS + umbilicus–ASIS) + 1.6	[18]
Verheij et al.'s formula (2013)	UVC length (cm) = [3 × BW (kg) + 9]/2	No proposed formula for UAC	[15]
Gupta et al.'s formula (2015)	UVC length (cm) = UN–1	UAC length (cm) = (UN − 1) + 2USp	[16]

Abbreviations: UVC: umbilical venous catheter; UAC: umbilical arterial catheter; UN: umbilical-nipple length; ASIS: anterior superior iliac spine; USp: the distance from the umbilicus to symphysis pubis.

**Table 2:** Endotracheal tube size of neonates.

Weight (g)	Gestational age (weeks)	ETT Size, inside diameter (mm)
<1000	<28	2.5
1000-2000	28-34	3.0
>2000	>34	3.5

**Table 3:** Methods of estimation of the proper insertion depth of endotracheal tube.

Method	ETT insertion depth	Reference
Rule of 7-8-9	7 cm for infants weighing 1 kg, 8 cm for infants weighing 2 kg, 9 cm for infants weighing 3 kg.	[96]
Nasal-tragus length (NTL)	The distance from the tip of the nasal septum to the tragus of the ear + 1 cm	[99]
The American Academy of Paediatrics/American Heart Association formula	Insertion depth (cm) = weight in kilograms + 6	[100]

**Table 4:** Recommended ETT length.

ETT length at lips (cm)	Corrected gestation (weeks)	Actual weight (kg)
5.5	23-24	0.5-0.6
6.0	25-26	0.7-0.8
6.5	27-29	0.9-1.0
7.0	30-32	1.1-1.4
7.5	33-34	1.5-1.8
8.0	35-37	1.9-2.4
8.5	38-40	2.5-3.1
9.0	41-43	3.2-4.2

**Table 5:** Minimum insertion length for an orogastric tube in very low-birth-weight infants.

Weight (g)	Insertion length of OGT (cm)
<750	13
750–999	15
1000–1249	16
1250–1499	17

**Table 6:** Nasogastric tube insertion length based on an age-related, height-based (ARHB) equation.

Neonate's length (cm)	NG tube insertion length (cm)
35.0-35.5	15.0
36.0-37.0	15.5
37.5-38.0	16.0
38.5-39.5	16.5
40.0-41.0	17.0
41.5-42.0	17.5
42.5-43.5	18.0
44.0-45.0	18.5
45.5-46.5	19.0
47.0-47.5	19.5
48.0-49.0	20.0
49.0-50.5	20.5
51.0-51.5	21.0
52.0-53.0	21.5
53.5-54.5	22.0
55.0-55.5	22.5
56.0-56.5	23.0

**Table 7:** Insertion depth of lumber puncture in neonates.

Insertion depth of lumber puncture	Reference
1.0-1.5 cm	[172]
0.03 × height of child (cm)	[173]
[13.19 + 0.0026 × weight (g) − 0.12 × postconceptual age in weeks] (mm)	[174]
[2 × weight (kg) + 7] (mm)	[175]
[2.5 × weight (kg) + 6] (mm)	[176]
